# Risk of stroke and bleeding in patients with heart failure and chronic kidney disease: a nationwide cohort study

**DOI:** 10.1002/ehf2.12256

**Published:** 2018-01-31

**Authors:** Line Melgaard, Thure Filskov Overvad, Flemming Skjøth, Jeppe Hagstrup Christensen, Torben Bjerregaard Larsen, Gregory Y.H. Lip

**Affiliations:** ^1^ Aalborg Thrombosis Research Unit, Department of Clinical Medicine, Faculty of Health Aalborg University Aalborg Denmark; ^2^ Department of Nephrology Aalborg University Hospital Aalborg Denmark; ^3^ Department of Cardiology, Thrombosis and Drug Research Unit Aalborg University Hospital Søndre Skovvej 15 DK‐9000 Aalborg Denmark; ^4^ Institute of Cardiovascular Sciences University of Birmingham Birmingham UK

**Keywords:** Heart failure, Chronic kidney disease, Stroke, Bleeding, Mortality

## Abstract

**Aims:**

The aim of this study was to assess the prognostic value of chronic kidney disease (CKD) in relation to ischaemic stroke, intracranial haemorrhage, major bleeding, and all‐cause death in heart failure patients without atrial fibrillation.

**Methods and results:**

In this observational cohort study, heart failure patients without atrial fibrillation were identified using Danish nationwide registries. Risk of stroke, major haemorrhage, and death were calculated after 1 and 5 years to compare patients with and without CKD, ±dialysis [dialysis: CKD with renal replacement therapy (CKD‐RRT); no dialysis: CKD‐no RRT]. A total of 43 199 heart failure patients were included, among which 0.8% had CKD‐RRT and 5.9% had CKD‐no RRT. When compared with heart failure patients without CKD, both CKD‐RRT and CKD‐no RRT were associated with a higher 5 year rate of major bleeding (CKD‐RRT: adjusted hazard ratio (aHR): 2.91, 95% confidence interval (CI): 2.29 to 3.70; CKD‐no RRT: aHR: 1.28, 95% CI: 1.13 to 1.45) and all‐cause death (CKD‐RRT: aHR: 2.40, 95% CI: 2.07 to 2.77; CKD‐no RRT: aHR: 1.63, 95% CI: 1.55 to 1.73). For the endpoints of ischaemic stroke and intracranial bleeding, only CKD‐no RRT was associated with significantly higher 5 year rates (ischaemic stroke: aHR: 1.31, 95% CI: 1.13 to 1.52; intracranial haemorrhage: aHR: 1.66, 95% CI: 1.04 to 2.65).

**Conclusions:**

Compared with patients without CKD, among incident heart failure patients without atrial fibrillation, CKD both with and without dialysis was associated with a higher rate of major bleeding and all‐cause death. Only CKD‐no RRT was associated with a higher rate of ischaemic stroke and intracranial bleeding.

## Introduction

Heart failure (HF) and chronic kidney disease (CKD) often coexist.^1^, ^2^, ^3^ Among HF patients with concomitant CKD, mortality and morbidity are high.^4^, ^5^ Nonetheless, there are limited data on the risk of stroke and bleeding, especially among HF patients without atrial fibrillation (AF).

In the HF population, thromboembolic risk stratification is evolving to help identify ‘high‐risk’ subjects, who may be targeted for more regular review and follow‐up, as well as more intensified cardiovascular prevention strategies.^6^ Chronic kidney disease has been associated with an increased risk of stroke in the general population,^7^ but whether CKD is a prognostic factor of stroke in patients with HF has not been investigated. Also, CKD has been associated with an increased risk of haemorrhagic stroke and major bleeding in patients with cardiovascular disease,^8^, ^9^ although this has not been specifically described in the HF population. As patients with CKD are a heterogeneous group, it may be necessary to subdivide these patients according to disease severity, e.g. CKD requiring dialysis, for optimal assessment of risks and clinical risk stratification.

The objective of the present observational cohort study was to assess the prognostic value of CKD in relation to the risk of ischaemic stroke, intracranial haemorrhage, major bleeding, and all‐cause death in HF patients without AF, using Danish nationwide administrative registries. We hypothesized that, compared with patients without CKD, in a population of incident HF patients without AF, CKD {both CKD with dialysis [CKD with renal replacement therapy (CKD‐RRT)] and without dialysis [CKD‐no RRT]} would be associated with a higher risk of ischaemic stroke, intracranial haemorrhage, major bleeding, and all‐cause death, also when controlling for concomitant cardiovascular risk factors.

## Methods

### Registry data sources

We used data from three nationwide registries: (i) the Danish National Patient Registry,^10^ which has registered all hospital admissions along with diagnoses since 1977 and codes all diagnoses according to the 10th revision of the *International Classification of Diseases* (ICD‐10) since 1994; (ii) the National Prescription Registry,^11^ which contains data on all prescriptions dispensed from Danish pharmacies since 1994, coded according to the Anatomical Therapeutic Chemical Classification System; and (iii) the Danish Civil Registration System, which holds information on date of birth, migration, vital status, date of death, and sex of all persons living in Denmark.^12^ Data were linked via a unique personal identification number used across all Danish national registries. Information from the three registries was retrieved until 31 December 2014. These registries have previously been validated,^10^, ^11^, ^12^ and the diagnoses of HF, stroke, and CKD were all found to have high validity.^13^, ^14^, ^15^

### Study population

The study population was identified as inpatient or outpatients aged >50 years, diagnosed with a primary discharge diagnosis of incident (first‐time diagnosis) HF in the period 1 January 2000 to 31 July 2014. Chronic kidney disease was defined as a diagnosis of CKD‐RRT (defined as a diagnosis of CKD and a concomitant procedure code for dialysis) or CKD‐no RRT (defined as a diagnosis of CKD and no concomitant procedure code for dialysis) between 1994 and time of HF diagnosis (for ICD‐10 codes used in the definitions of CKD‐RRT and CKD‐no RRT, see *Table* *S1*). Chronic kidney disease status was assessed at the time of the HF diagnosis, and status could not change throughout the follow‐up period. To restrict to patients without AF, we excluded those who had a prior diagnosis of AF or atrial flutter between 1994 and time of HF diagnosis. We excluded patients treated with a vitamin K antagonist within 6 months prior to the HF diagnosis to avoid considering effect modification by anticoagulation therapy, and because such patients may have AF that is diagnosed but unregistered. Additionally, we excluded patients with a prior procedure code for kidney transplantation, as this small patient group may be difficult to categorize with the available data from the registries. Lastly, patients with a diagnosis of cancer within 5 years before HF diagnosis were excluded, because cancer patients represents a subgroup with high stroke risk^16^ and specialized thromboprophylactic treatment regimens.

Additional co‐morbidities were assessed at time of HF diagnosis and identified using the Danish National Patient Registry and the Danish National Prescription Registry. Ascertainment of baseline medication status was based on medication purchase in a 45 day window before or after the date of HF diagnosis to express the status in the follow‐up period. ICD codes and Anatomical Therapeutic Chemical codes used to define co‐morbidities and medical therapy are provided in *Table* *S1*.

### Outcomes

The primary endpoints were defined as a diagnosis of ischaemic stroke or bleeding. Bleeding was included as intracranial haemorrhage and major bleeding (combined endpoint of intracranial haemorrhage, gastrointestinal bleeding, and extracranial or unclassified major bleeding). All‐cause death was also included as an outcome, because of the high mortality among HF patients and the appertaining possibility of distortion from competing risks, and the fact that in administrative registries some deaths may be due to undiagnosed stroke or bleeding.

### Statistical methods

Baseline characteristics were described separately for patients without CKD, CKD‐no RRT, and CKD‐RRT using means and standard deviation for continuous variables, and proportions for categorical variables. Time‐to‐event analysis was used to describe the association between CKD status (CKD‐no RRT or CKD‐RRT) and the risk of ischaemic stroke, intracranial haemorrhage, major bleeding, or all‐cause death. Time at risk was measured from baseline date (date of HF diagnosis) and until an event of ischaemic stroke, intracranial haemorrhage, major bleeding, all‐cause death, emigration, or end of study (31 December 2014), whichever came first. Additionally, patients were censored if they initiated anticoagulant therapy during the follow‐up period.

Crude cumulative incidence curves for all endpoints according to CKD status (no CKD, CKD‐no RRT, or CKD‐RRT) were constructed based on the Aalen–Johansen estimator^17^ for competing risk data. We have previously advocated risks (probabilities) rather than rates for assessing associations in an HF population, as risks lead to statements of greater clinical and prognostic relevance when faced with a high competing mortality risk.^18^ However, strong differential competing mortality across exposure levels can lead to counterintuitive findings on a risk scale, which is a major concern for CKD as an exposure. Therefore, we reported associations in terms of (Cox model) hazard ratios (HRs) after 1 and 5 years of follow‐up. Following the suggestions by Andersen *et al*.^19^ of considering both risk and rate assessments, we repeated the analysis on a risk (ratio) scale; see the Supporting Information for methodological details.

For the main analysis, Cox regression was used to calculate 1 and 5 year HRs of the endpoints according to the status of CKD (CKD‐no RRT or CKD‐RRT), with patients without CKD as reference group. Concomitant baseline co‐morbidities such as hypertension, diabetes, vascular disease, and prior ischaemic stroke are well‐known prognostic factors of stroke and bleeding in other heart diseases.^20^, ^21^ Thus, we fitted the Cox models after adjusting for these clinical factors, including age and sex. These prognostic factors are also included in the CHA_2_DS_2_‐VASc [congestive HF, hypertension, age ≥ 75 years (doubled), diabetes, stroke/transient ischaemic attack/thromboembolism (doubled), vascular disease (prior myocardial infarction, peripheral artery disease, or aortic plaque), age 65–75 years, sex category (female)] score; thus, we basically adjusted for components of this score. Additionally, as antiplatelet therapy may modify the association between CKD and risk of the endpoints, we also included use of antiplatelet therapy in the main adjusted Cox model.

Analyses were performed using Stata version 13 (Stata Corporation, College Station, TX, USA). Results are reported with 95% confidence intervals (CIs).

### Supplementary and sensitivity analyses

As some patients might have a diagnosis of AF shortly after the HF diagnosis, a sensitivity analysis was performed by repeating the HR calculations after extending the definition of concomitant AF at baseline; AF patients were identified if they had a prior diagnosis code for AF at baseline (at time of HF diagnosis) or received a diagnosis code for AF within 30 days after the HF diagnosis. Furthermore, some patients are diagnosed with AF during follow‐up; thus, to investigate whether the inclusion of AF patients affects our main results, we performed another sensitivity analysis after censoring patients who are diagnosed with AF during follow‐up.

In a supplementary analysis, to investigate whether the chosen well‐known risk factors are correctly included in the main adjusted model, additional models were constructed adjusting for age and sex only (model 1) and age, sex, and each individual risk factors (Models 2–6). The results of these additional models are presented in the Supporting Information.

### Ethical considerations

No ethical approval is required for studies based on data from administrative Danish registries. The study was approved by the Danish Data Protection Agency (J. No. File No. 2015‐57‐0001). The study was conducted and reported in accordance with the STROBE (Strengthening the Reporting of Observational Studies in Epidemiology) recommendations.

## Results

**Figure 1:**
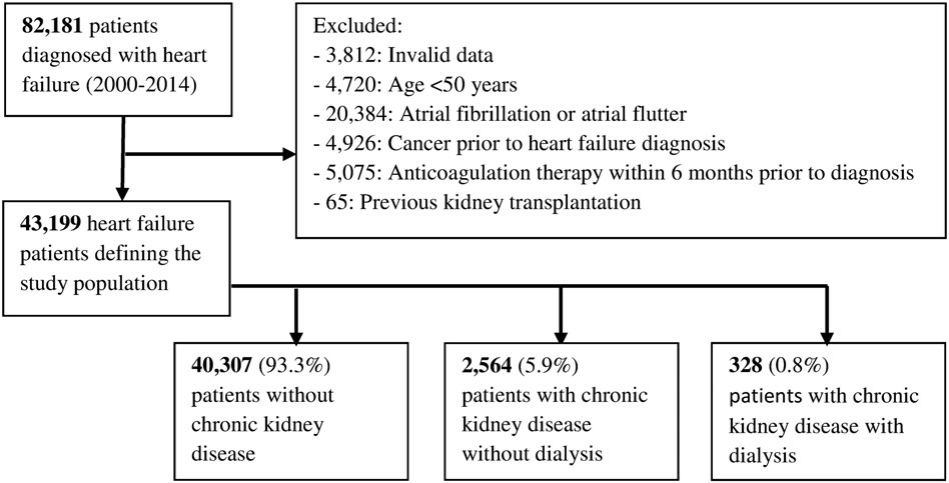
Flowchart of patients included in the final study population.

The study population comprised 43 199 HF patients aged >50 years, among which 0.8% had CKD‐RRT and 5.9% had CKD‐no RRT at baseline (*Figure* *1*). The median follow‐up time for ischaemic stroke or all‐cause death was 2.3 years (inter‐quartile range 0.5 to 5.3).

Baseline patient characteristics are summarized in *Table* 1. Mean age was similar among HF patients without CKD and CKD‐no RRT, but lower for patients with CKD‐RRT. Patients with CKD had more prevalent prior stroke/transient ischaemic attack and major bleeding than did patients without CKD; also, the prevalence of diabetes, hypertension, and vascular disease was higher in patients with CKD, as expected.

**Table 1:** Baseline characteristics of study population according to chronic kidney disease status

Clinical characteristics	No chronic kidney disease	Chronic kidney disease without dialysis	Chronic kidney disease with dialysis
*n*, % (*n*)	93.3 (40 307)	5.9 (2564)	0.8 (328)
Sex (female), % (*n*)	44.5 (17 947)	37.6 (964)	33.8 (111)
Mean age, years (SD)	73.9 (11.4)	75.1 (10.9)	69.0 (9.0)
Co‐morbidity, % (*n*)			
Previous ischaemic stroke/transient ischaemic attack	12.6 (5082)	20.1 (515)	19.5 (64)
Previous intracranial haemorrhage	1.1 (460)	1.1 (27)	1.5 (5)
Previous major bleeding	18.3 (7375)	32.3 (827)	37.5 (123)
Diabetes	12.8 (5140)	37.8 (970)	32.3 (106)
Hypertension	30.8 (12 416)	61.4 (1574)	72.0 (236)
Vascular disease	30.7 (12 384)	43.1 (1104)	45.4 (149)
Previous myocardial infarction	25.3 (10 180)	31.0 (794)	30.8 (101)
Liver disease	0.4 (172)	1.2 (30)	1.2 (4)
Hyperthyroidism	2.6 (1065)	2.6 (66)	3.4 (11)
COPD	13.4 (5413)	16.5 (424)	11.0 (36)
Medication, % (*n*)			
ACE‐inhibitors	53.3 (21 498)	39.5 (1014)	39.9 (131)
Angiotensin receptor blocker	10.7 (4317)	17.4 (446)	16.8 (55)
Beta‐blockers	45.9 (18 480)	47.9 (1228)	57.9 (190)
Aldosterone antagonists	23.5 (9475)	18.4 (471)	3.7 (12)
Non‐loop diuretics	39.6 (15 975)	33.3 (854)	6.1 (20)
Loop diuretics	65.0 (26 182)	74.8 (1917)	58.2 (191)
Statins	32.4 (13 068)	37.9 (973)	31.1 (102)
NSAIDs	13.8 (5577)	13.5 (346)	4.0 (13)
Aspirin	48.6 (19 586)	49.0 (1256)	38.7 (127)
Thienopyridines	12.3 (4960)	12.6 (323)	13.7 (45)

ACE, angiotensin‐converting‐enzyme; COPD, chronic obstructive pulmonary disease; NSAIDs, non‐steroidal anti‐inflammatory drugs; SD, standard deviation.

**Figure 2:**
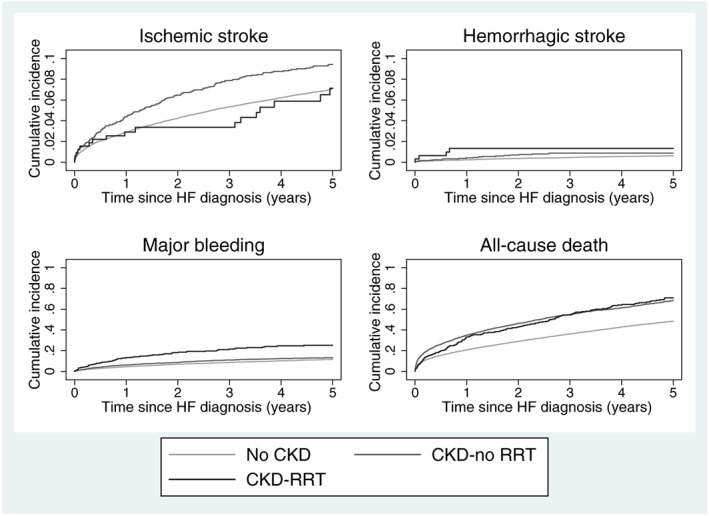
Crude cumulative incidence of all endpoints according to chronic kidney disease status (note the varying range of the *y*‐axes). Abbreviations: CKD, chronic kidney disease; CKD‐RRT, chronic kidney disease with dialysis; CKD‐no RRT, chronic kidney disease without dialysis; HF, heart failure.

The number of events and the absolute risk of ischaemic stroke, intracranial haemorrhage, major bleeding, and all‐cause death in each patient group after 1 and 5 years of follow‐up are shown in *Table* 2. For patients with CKD‐RRT, the absolute risk of all endpoints was high, after both 1 and 5 years of follow‐up (ischaemic stroke: 2.5 and 6.9; intracranial haemorrhage: 1.3 and 1.3; major bleeding: 13.0 and 24.9; all‐cause death: 32.8 and 69.7, respectively). Notably, the risk of all‐cause death was very high in all patient groups after 5 years of follow‐up (48.5%, 68.2%, and 69.7%, for patients without CKD, CKD‐no RRT, and CKD‐RRT, respectively). The number of intracranial haemorrhage events was low in our study population. Crude cumulative incidence curves for all endpoints demonstrate a steady increase during follow‐up, as seen in *Figure* *2*.

**Table 2:** Event numbers and absolute risks of all endpoints after 1 and 5 years of follow‐up, according to chronic kidney disease status

Endpoint	1 year of follow‐up		5 years of follow‐up	
	No. of events	Absolute risk^a^ (%)	No. of events	Absolute risk^a^ (%)
Ischaemic stroke				
No chronic kidney disease	1112	2.9	2391	7.1
Chronic kidney disease without dialysis	107	4.4	205	9.4
Chronic kidney disease with dialysis	8	2.5	17	6.9
Intracranial haemorrhage				
No chronic kidney disease	89	0.2	214	0.7
Chronic kidney disease without dialysis	10	0.4	21	1.0
Chronic kidney disease with dialysis	4	1.3	4	1.3
Major bleeding^b^				
No chronic kidney disease	1728	4.6	3905	11.7
Chronic kidney disease without dialysis	152	6.3	289	13.4
Chronic kidney disease with dialysis	40	13.0	69	24.9
All‐cause death				
No chronic kidney disease	7944	20.8	16 290	48.5
Chronic kidney disease without dialysis	860	35.0	1487	68.2
Chronic kidney disease with dialysis	101	32.8	187	69.7

a Taking into account competing risks of death (Aalen–Johansen estimator).

b Combined endpoint of intracranial haemorrhage, gastrointestinal bleeding, extracranial, or unclassified major bleeding.

### Multivariable analysis

**Figure 3:**
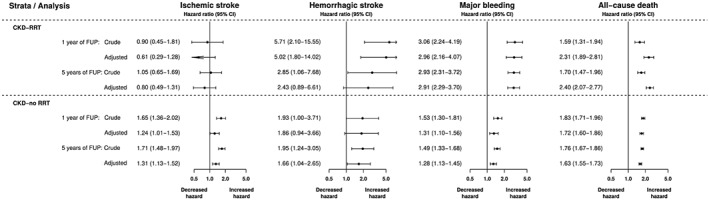
Hazard rate ratios of all endpoints after 1 and 5 years of follow‐up, according to chronic kidney disease status (reference group: patients without chronic kidney disease). Abbreviations: 95% CI, 95% confidence interval; FUP, follow‐up. Analysis adjusted for sex (binary), age (continuous), hypertension (binary), diabetes (binary), prior stroke/transient ischaemic attack (binary), vascular disease (binary), and antiplatelet therapy (binary).

In the Cox regression analysis adjusted for concomitant prognostic factors, when compared with HF patients without CKD, both CKD‐RRT and CKD‐no RRT were associated with a higher 5 year rate of major bleeding (CKD‐RRT: adjusted HR: 2.91, 95% CI: 2.29 to 3.70; CKD‐no RRT: adjusted HR: 1.28, 95% CI: 1.13 to 1.45) and all‐cause death (CKD‐RRT: adjusted HR: 2.40, 95% CI: 2.07 to 2.77; CKD‐no RRT: adjusted HR: 1.63, 95% CI: 1.55 to 1.73; *Figure* *3*). For the endpoints of ischaemic stroke and intracranial bleeding, only CKD‐no RRT was associated with significantly higher 5 year rates (ischaemic stroke: adjusted HR: 1.31, 95% CI: 1.13 to 1.52; intracranial haemorrhage: adjusted HR: 1.66, 95% CI: 1.04 to 2.65).

Similar results were obtained after 1 year of follow‐up.

The 1 and 5 year relative risks of all endpoints, comparing patients with no CKD, CKD‐no RRT, and CKD‐RRT, are shown in *Tables S2* and *S3*. We found similar, although slightly lower, crude associations as in the rate‐based calculations, but the associations attenuated after adjustment for other cardiovascular prognostic factors, especially for the ischaemic stroke endpoint.

### Supplementary and sensitivity analyses

In the sensitivity analysis excluding patients with an AF diagnosis at baseline or within 30 days after the HF diagnosis, findings were similar to those of the main analysis (*Table S4*). In the sensitivity analysis in which patients were censored if they were diagnosed with AF during follow‐up, we found similar results as in the main analysis (*Table S5*).

## Discussion

In this nationwide cohort study of HF patients without diagnosed AF and not taking anticoagulant therapy, we found higher rates of intracranial haemorrhage, major bleeding, and all‐cause death among HF patients with CKD‐RRT and CKD‐no RRT compared with patients without CKD, even after extensive adjustment for concomitant cardiovascular risk factors. For the endpoint ischaemic stroke, only CKD‐no RRT was associated with a higher risk.

To our knowledge, this is the first study to evaluate the prognostic value of CKD in relation to ischaemic stroke, intracranial haemorrhage, major bleeding, and all‐cause death in an HF population without diagnosed AF.

The current evidence on the risk of stroke in subgroups of HF patients without AF is very limited.^22^, ^23^, ^24^ Our finding of an increased risk of ischaemic stroke in patients with CKD‐no RRT is similar to previous findings from non‐HF populations.^9^, ^25^ As the aim of our study was to examine the *prognostic value* of CKD in relation to stroke, major bleeding, and mortality in HF, we can only speculate on the aetiological explanations for our observations. For example, the increased stroke risk associated with CKD may be explained by the coexistence of platelet dysfunction, endothelial damage/dysfunction, and prothrombotic and inflammatory state, which is often seen in patients with CKD as well as in HF.^26^ Additionally, the increased risk of bleeding may be due to platelet dysfunction, prolonged bleeding time, and small vessel disease associated with CKD.^27^, ^28^ Overall, CKD may be associated with adverse outcomes in HF, as it is a marker of more severe HF, greater symptom burden, and/or coexistent disease.^1^, ^2^, ^29^ The poorer survival in patients with CKD and HF may also reflect the reduced likelihood of being prescribed evidence‐based therapies,^29^, ^30^ as CKD is often viewed as a contraindication to some therapies,^2^, ^29^ including thromboprophylactic therapies.^31^ This is correspondingly reflected in randomized trials testing the efficacy and safety of therapies, where patients with CKD are often excluded, resulting in clinicians being challenged in how to choose the optimal treatment for these patients.

With the focus on refining risk stratification, our findings indicate that CKD may be an important prognostic factor in high‐risk patients with HF without AF. However, in the present study, only CKD‐no RRT was associated with an increased risk of ischaemic stroke. This probably reflects the fact that patients with CKD‐RRT are on dialysis (and receive low‐molecular‐weight heparin), which may reduce their thrombotic risk. Thus, in patients with CKD and HF, individualized risk assessment according to disease severity is necessary to optimize cardiovascular prevention strategies.

We provided both risk/probability (risk ratio) and rate (HR) assessments of the associations.^19^ While risk and rate assessments are traditionally thought of as being equivalent, they can be fundamentally different in the face of competing mortality risk.^32^ In the present study, associations were attenuated when viewed on a risk scale. This is important information from a clinical perspective, as it may indicate a smaller absolute potential of prevention strategies among patients with CKD and HF than otherwise suggested by the HRs.

### Strengths and limitations

The large sample size uniquely possible with this type of cohort study minimizes the risk of random error. Selection into the study was not an issue, as we investigated a nationwide population cohort of incident HF patients without AF using administrative data, which also implies very limited loss to follow‐up.

The diagnosis of HF has previously been validated with a sensitivity of 29%, a specificity of 99%, and a positive predictive value of 81–100%^15^, ^33^; thus, we likely did not capture all patients with HF and also cannot be certain that all patients identified as having HF had definite HF. Nonetheless, this most likely applies equally to both exposed and non‐exposed, and the suboptimal positive predictive value is therefore not a likely explanation for the observed associations. Also, we included only patients with a primary discharge diagnosis of HF to optimize the probability of including only correctly identified patients with HF.

Patients with CKD were identified using ICD‐10 codes in the Danish registries, which have also been used in previous registry‐based studies of patients with CKD.^9^, ^25^ However, we did not have access to biomarkers, and therefore, CKD could be under‐reported, as a measurement outside normal ranges might not necessarily trigger a diagnosis in the registries. We cannot rule out that some patients might have had undiagnosed AF, because (any) heart disease is associated with an increased risk of developing AF; however, censoring for a diagnosis of AF during follow‐up did not change our main conclusions. The diagnosis of ischaemic stroke has also been validated and found to have a positive predictive value of 80–90%.^13^, ^34^ We included unspecified stroke in the definition of ischaemic stroke, as many such strokes are of ischaemic origin.^13^ However, we cannot rule out that some of these strokes were haemorrhagic strokes and, thus, misclassified as ischaemic strokes. Nonetheless, such issues with diagnostic coding are likely to apply equally among exposed and non‐exposed, and our findings are, therefore, unlikely to be explained by information bias.

We investigated whether the presence of CKD was associated with ischaemic stroke, intracranial haemorrhage, major bleeding, and all‐cause death in patients with HF, and therefore, we adjusted for well‐known cardiovascular prognostic factors for stroke and bleeding. This was not an attempt to adjust for confounding and hereby explore the potential causal relationship between the exposure and outcomes, but to elucidate the potential added predictive ability of the exposure in relation to risk stratification of patients with HF after adjustment for other possible prognostic factors, essentially those included in the previously investigated CHA_2_DS_2_‐VASc score.^6^ As the focus was on the prognostic value of a diagnosis of CKD in relation to stroke and bleeding, not its causal role, potential confounding by other stroke and bleeding risk factors (e.g. blood pressure level, smoking, body mass index, and lipid status) is of lesser concern in this study. Additionally, as the focus of the study was on the prognostic value in incident HF patients, a change in CKD status during follow‐up was less important. The data used in this study were not collected for the primary purpose of this study, which is a limitation with respect to missing information about selected variables that could have been of interest, e.g. lifestyle factors. However, the Danish nationwide registries have been acknowledged as a valuable tool and data source for epidemiological research.^35^, ^36^

### Generalizability of the study results

The study population consisted only of patients aged >50 years, and HF in persons aged <50 years might represent a different group of patients, for example, patients with congenital heart disease. Accordingly, our findings may not apply to younger HF patients. Additionally, we were unable to distinguish between HF with preserved and reduced ejection fraction or to estimate the functional classification, as we did not have access to echocardiograms, so we cannot be sure that the results apply to patients with preserved and reduced ejection fraction. Lastly, the study was carried out as a nationwide study in the Danish population, which ethnically is fairly homogeneous; thus, future studies are needed to evaluate if our findings hold in more ethnically diverse populations.

In conclusion, compared with patients without CKD, among incident HF patients without AF, CKD, despite severity, was associated with a higher rate of major bleeding and all‐cause death. Only CKD‐no RRT was associated with higher rates of ischaemic stroke and intracranial bleeding. Chronic kidney disease may have prognostic value when considering cardiovascular prevention strategies in the HF population without AF.

## Conflict of interest

All authors have completed the ICMJE uniform disclosure form at http://www.icmje.org/coi_disclosure.pdf and declare the following.

G.Y.H.L. is consultant for Bayer/Janssen, BMS/Pfizer, Biotronik, Medtronic, Boehringer Ingelheim, Microlife, and Daiichi‐Sankyo; and speaker for Bayer, BMS/Pfizer, Medtronic, Boehringer Ingelheim, Microlife, Roche, and Daiichi‐Sankyo. No fees are received personally.

T.B.L. is an investigator for Janssen Scientific Affairs, LLC, and Boehringer Ingelheim; and speaker for Bayer, BMS/Pfizer, Janssen Pharmaceuticals, Takeda, Roche Diagnostics, and Boehringer Ingelheim. No fees are received personally.

All other authors declare no conflict of interest.

Statistics Denmark provided the data for this study. No additional data are available beside the Supporting Information.

## Supporting information


**Table S1**. ICD10‐codes and ATC‐codes used in the study.
**Table S2**. Relative risks of all endpoints after 1 year of follow‐up, according to chronic kidney disease status (reference group: patients without chronic kidney disease).
**Table S3**. Relative risks of all endpoints after 5 years of follow‐up, according to chronic kidney disease status (reference group: patients without chronic kidney disease).
**Table S4**. Sensitivity analysis excluding patients with an atrial fibrillation diagnosis at baseline or within 30 days after the heart failure diagnosis: Hazard rate ratios of all endpoints after 5 years of follow‐up, according to chronic kidney disease status (reference group: patients without chronic kidney disease).
**Table S5**. Sensitivity analysis censoring patients diagnosed with atrial fibrillation during follow‐up: Hazard rate ratios of all endpoints after 5 years of follow‐up, according to chronic kidney disease status (reference group: patients without chronic kidney disease).
**Table S6**. Hazard rate ratios of all endpoints after 1 year of follow‐up (using different adjusted models), according to chronic kidney disease status (reference group: patients without chronic kidney disease).
**Table S7**. Hazard rate ratios of all endpoints after 5 years of follow‐up (using different adjusted models), according to chronic kidney disease status (reference group: patients without chronic kidney disease).
