# Supplementary material for: Risk of stroke and bleeding in patients with heart failure and chronic kidney disease: a nationwide cohort study
Source: ESC Heart Fail. 2018 Jan 31;5(2):319–26. doi: 10.1002/ehf2.12256 (PMC5880668; doi:10.1002/ehf2.12256)
Supplement: Supplementary file 1 — Table S1. ICD10‐codes and ATC‐codes used in the study. Table S2. Relative risks of all endpoints after 1 year of follow‐up, according to chronic kidney disease status (reference group: patients without chronic kidney disease). Table S3. Relative risks of all endpoints after 5 years of follow‐up, according to chronic kidney disease status (reference group: patients without chronic kidney disease). Table S4. Sensitivity analysis excluding patients with an atrial fibrillation diagnosis at baseline or within 30 days after the heart failure diagnosis: Hazard rate ratios of all endpoints after 5 years of follow‐up, according to chronic kidney disease status (reference group: patients without chronic kidney disease). Table S5. Sensitivity analysis censoring patients diagnosed with atrial fibrillation during follow‐up: Hazard rate ratios of all endpoints after 5 years of follow‐up, according to chronic kidney disease status (reference group: patients without chronic kidney disease). Table S6. Hazard rate ratios of all endpoints after 1 year of follow‐up (using different adjusted models), according to chronic kidney disease status (reference group: patients without chronic kidney disease). Table S7. Hazard rate ratios of all endpoints after 5 years of follow‐up (using different adjusted models), according to chronic kidney disease status (reference group: patients without chronic kidney disease). [file EHF2-5-319-s001.docx]

**SUPPLEMENTARY FILE**

**e-Table 1**. ICD10-codes and ATC-codes used in the study.

**e-Table 2**. Relative risks of all endpoints after 1 year of follow-up, according to chronic kidney disease status (reference group: patients without chronic kidney disease).

**e-Table 3**. Relative risks of all endpoints after 5 year of follow-up, according to chronic kidney disease status (reference group: patients without chronic kidney disease).

**e-Table 4**. Sensitivity analysis excluding patients with an atrial fibrillation diagnosis at baseline or within 30 days after the heart failure diagnosis: Hazard rate ratios of all endpoints after 5 years of follow-up, according to chronic kidney disease status (reference group: patients without chronic kidney disease).

**e-Table 5**. Sensitivity analysis censoring patients diagnosed with atrial fibrillation during follow-up: Hazard rate ratios of all endpoints after 5 years of follow-up, according to chronic kidney disease status (reference group: patients without chronic kidney disease).

**e-Table 6**. Hazard rate ratios of all endpoints after 1 year of follow-up (using different adjusted models), according to chronic kidney disease status (reference group: patients without chronic kidney disease).

**e-Table 7**. Hazard rate ratios of all endpoints after 5 years of follow-up (using different adjusted models), according to chronic kidney disease status (reference group: patients without chronic kidney disease).

**Methodological details.** Analysis on a risk-scale.

**e-Table 1**. ICD 10-codes and ATC-codes used in the study.

| **ICD 10-Codes and ATC-Codes used in the Study** | |
| --- | --- |
| **Main diagnosis** | **ICD 10-Codes** |
| Congestive heart failure | I50.0-I50.9, I11.0, I13.0, I13.2 |
| Chronic kidney disease without dialysis | I12.0-I12.9, I13.0-I13.9, N00-N05, N07-N08, N11-N12, N14.0-N14.4, N15.8, N16.0, N16.2-N16.4, N16.8, N17.0-N17.9, N18.0-N18.9, N19, Q61.0-Q61.9, E10.2, E11.2, E13.2, E14.2 |
| Chronic kidney disease with dialysis | I12.0-I12.9, I13.0-I13.9, N00-N05, N07-N08, N11-N12, N14.0-N14.4, N15.8, N16.0, N16.2-N16.4, N16.8, N17.0-N17.9, N18.0-N18.9, N19, Q61.0-Q61.9, E10.2, E11.2, E13.2, E14.2 and BJFD2 |
| **Endpoints** | **ICD 10-Codes** |
| Ischemic stroke | I63, I64 |
| Intracranial hemorrhage | I60-I62 |
| Major bleeding | I60-I62, K25-K29, S063C, S064-S066, D62, J942, H113, H356, H431, N02, R04, R31, R58 |
| **Comorbidities** | **ICD 10-Codes** |
| Prior ischemic stroke or transient ischemic attack | I63.0-I63.9, I64.9, G45 |
| Prior intracranial hemorrhage | I60-I62 |
| Prior major bleeding | I60-I62, K25-K29, S063C, S064-S066, D62, J942, H113, H356, H431, N02, R04, R31, R58 |
| Diabetes mellitus | E10.0, E10.1, E10.9, E11.0, E11.1, E11.9 |
| Hypertension | I10.0-I10.9, I11.0-I11.9, I12.0-I12.9, I13.0-I13.9, I15.0–I15.9 |
| Vascular disease | I21.0-I21.9, I23.0-I23.9, I70.2-I70.9, I71, I73.9 |
| Prior myocardial infarction | I21.0-I21.9, I23.0-I23.9 |
| Liver disease | B15.0, B16.0, B16.2, B19.0, K70.4, K72.0-K72.9, K76.6, I85 |
| Hyperthyroidism | E05.0-E05.9, E06.0-E06.9 |
| Chronic obstructive pulmonary disease (COPD) | J40-J47, J60-J65, J67, J68.4, J70.1, J70.3, J84.1, J92.0, J96.1, J98.2, J98.3 |
| Atrial fibrillation and flutter (exclusion criterion) | I48 |
| Cancer any type (exclusion criterion) | C00-C97 |
| **Concomitant medication** | **ATC-Codes** |
| Warfarin (exclusion criterion) | B01AA03 |
| Phenprocoumon (exclusion criterion) | B01AA04 |
| ACE-inhibitors | C09AA |
| Angiotensin receptor blockers | C09CA |
| Beta-blockers | C07 |
| Non-loop diuretics | C02DA, C02L, C03A, C03B, C03D, C03E, C03X, C07C, C07D, C08G,C09BA, C09DA, C09XA52 |
| Aldosterone antagonists | C03DA |
| Loop diuretics | C03C |
|  |  |
| Statins | C10 |
| Non steroidal anti-inflammatory drugs (NSAIDs) | M01A |
| Aspirin | B01AC06 |
| Thienopyridines | B01AC04, B01AC22, B01AC24 |

**e-Table 2**. Relative risks of all endpoints after 1 year of follow-up, according to chronic kidney disease status (reference group: patients without chronic kidney disease).

| ENDPOINT | | Chronic kidney disease without dialysis  (CKD-no RRT) | | Chronic kidney disease with dialysis (CKD-RRT) | |
| --- | --- | --- | --- | --- | --- |
|  | |  |  |  |  |
| Ischemic stroke | |  |  |  |  |
|  | Crude RR (95% CI) |  | 1.50 (1.24-1.83) |  | 0.87 (0.43-1.74) |
|  | Adjusted RR† (95% CI) |  | 0.98 (0.76-1.26) |  | 0.33 (0.11-0.93) |
|  |  |  |  |  |  |
| Intracranial hemorrhage | |  |  |  |  |
|  | Crude RR (95% CI) |  | 1.77 (0.92-3.41) |  | 5.63 (2.08-15.26) |
|  | Adjusted RR† (95% CI) |  | NA** |  | NA** |
|  |  |  |  |  |  |
| Major bleeding║ | |  |  |  |  |
|  | Crude RR (95% CI) |  | 1.37 (1.16-1.61) |  | 2.85 (2.12-3.82) |
|  | Adjusted RR† (95% CI) |  | 1.17 (0.98-1.39) |  | 2.66 (1.97-3.60) |
|  |  |  |  |  |  |
| All-cause death | |  |  |  |  |
|  | Crude RR (95% CI) |  | 1.69 (1.59-1.79) |  | 1.58 (1.34-1.86) |
|  | Adjusted RR† (95% CI) |  | 1.41 (1.33-1.49) |  | 1.94 (1.63-2.31) |
|  |  |  |  |  |  |
| Abbreviations: RR: relative risk; 95% CI: 95% confidence interval.  † Adjusted for sex (binary), age (continuous), hypertension (binary), diabetes (binary), prior stroke/transient ischemic attack (binary), vascular disease (binary), and antiplatelet therapy (binary).  ║Combined endpoint of intracranial hemorrhage, gastrointestinal bleeding, extracranial or unclassified major bleeding.  **Adjusted relative risks could not be estimated due to the very low event number of this rare outcome. | | | | | |

**e-Table 3**. Relative risks of all endpoints after 5 year of follow-up, according to chronic kidney disease status (reference group: patients without chronic kidney disease).

| ENDPOINT | | Chronic kidney disease without dialysis  (CKD-no RRT) | | Chronic kidney disease with dialysis (CKD-RRT) | |
| --- | --- | --- | --- | --- | --- |
|  | |  |  |  |  |
| Ischemic stroke | |  |  |  |  |
|  | Crude RR (95% CI) |  | 1.32 (1.15-1.52) |  | 0.96 (0.61-1.52) |
|  | Adjusted RR† (95% CI) |  | 0.99 (0.84-1.15) |  | 0.57 (0.33-0.99) |
|  |  |  |  |  |  |
| Intracranial hemorrhage | |  |  |  |  |
|  | Crude RR (95% CI) |  | 1.25 (1.01-2.57) |  | 2.16 (1.34-3.47) |
|  | Adjusted RR† (95% CI) |  | 1.47 (0.73-2.93) |  | 1.53 (0.51-4.62) |
|  |  |  |  |  |  |
| Major bleeding║ | |  |  |  |  |
|  | Crude RR (95% CI) |  | 1.14 (1.02-1.27) |  | 2.12 (1.72-2.60) |
|  | Adjusted RR† (95% CI) |  | 1.02 (0.91-1.15) |  | 1.97 (1.60-2.43) |
|  |  |  |  |  |  |
| All-cause death | |  |  |  |  |
|  | Crude RR (95% CI) |  | 1.41 (1.36-1.45) |  | 1.44 (1.32-1.56) |
|  | Adjusted RR† (95% CI) |  | 1.21 (1.18-1.25) |  | 1.63 (1.50-1.77) |
|  |  |  |  |  |  |
| Abbreviations: RR: relative risk; 95% CI: 95% confidence interval.  † Adjusted for sex (binary), age (continuous), hypertension (binary), diabetes (binary), prior stroke/transient ischemic attack (binary), vascular disease (binary), and antiplatelet therapy (binary).  ║Combined endpoint of intracranial hemorrhage, gastrointestinal bleeding, extracranial or unclassified major bleeding. | | | | | |

**e-Table 4**. Sensitivity analysis excluding patients with an atrial fibrillation diagnosis at baseline or within 30 days after the heart failure diagnosis: Hazard rate ratios of all endpoints after 5 years of follow-up, according to chronic kidney disease status (reference group: patients without chronic kidney disease).

| ENDPOINT | | Chronic kidney disease without dialysis  (CKD-no RRT) | | Chronic kidney disease with dialysis (CKD-RRT) | |
| --- | --- | --- | --- | --- | --- |
|  | |  |  |  |  |
| Ischemic stroke | |  |  |  |  |
|  | Crude HR (95% CI) |  | 1.70 (1.47-1.97) |  | 1.08 (0.67-1.75) |
|  | Adjusted HR† (95% CI) |  | 1.30 (1.11-1.51) |  | 0.84 (0.51-1.37) |
|  |  |  |  |  |  |
| Intracranial hemorrhage | |  |  |  |  |
|  | Crude HR (95% CI) |  | 1.72 (1.06-2.78) |  | 2.94 (1.09-7.90) |
|  | Adjusted HR† (95% CI) |  | 1.45 (0.88-2.39) |  | 2.50 (0.92-6.81) |
|  |  |  |  |  |  |
| Major bleeding║ | |  |  |  |  |
|  | Crude HR (95% CI) |  | 1.48 (1.31-1.67) |  | 2.95 (2.32-3.75) |
|  | Adjusted HR† (95% CI) |  | 1.27 (1.12-1.44) |  | 2.95 (2.31-3.76) |
|  |  |  |  |  |  |
| All-cause death | |  |  |  |  |
|  | Crude HR (95% CI) |  | 1.77 (1.68-1.87) |  | 1.69 (1.46-1.96) |
|  | Adjusted HR† (95% CI) |  | 1.63 (1.55-1.73) |  | 2.47 (2.13-2.86) |
|  |  |  |  |  |  |
| Abbreviations: HR: hazard rate ratio; 95% CI: 95% confidence interval.  † Adjusted for sex (binary), age (continuous), hypertension (binary), diabetes (binary), prior stroke/transient ischemic attack (binary), vascular disease (binary), and antiplatelet therapy (binary).  ║Combined endpoint of intracranial hemorrhage, gastrointestinal bleeding, extracranial or unclassified major bleeding. | | | | | |

**e-Table 5**. Sensitivity analysis censoring patients diagnosed with atrial fibrillation during follow-up: Hazard rate ratios of all endpoints after 5 years of follow-up, according to chronic kidney disease status (reference group: patients without chronic kidney disease).

| ENDPOINT | | Chronic kidney disease without dialysis  (CKD-no RRT) | | Chronic kidney disease with dialysis (CKD-RRT) | |
| --- | --- | --- | --- | --- | --- |
|  | |  |  |  |  |
| Ischemic stroke | |  |  |  |  |
|  | Crude HR (95% CI) |  | 1.71 (1.48-1.98) |  | 1.14 (0.71-1.83) |
|  | Adjusted HR† (95% CI) |  | 1.29 (1.11-1.51) |  | 0.86 (0.53-1.41) |
|  |  |  |  |  |  |
| Intracranial hemorrhage | |  |  |  |  |
|  | Crude HR (95% CI) |  | 1.87 (1.17-3.00) |  | 3.05 (1.13-8.21) |
|  | Adjusted HR† (95% CI) |  | 1.57 (0.97-2.56) |  | 2.56 (0.94-6.98) |
|  |  |  |  |  |  |
| Major bleeding║ | |  |  |  |  |
|  | Crude HR (95% CI) |  | 1.49 (1.32-1.69) |  | 2.97 (2.33-3.79) |
|  | Adjusted HR† (95% CI) |  | 1.27 (1.12-1.44) |  | 2.95 (2.31-3.78) |
|  |  |  |  |  |  |
| All-cause death | |  |  |  |  |
|  | Crude HR (95% CI) |  | 1.79 (1.70-1.90) |  | 1.67 (1.43-1.94) |
|  | Adjusted HR† (95% CI) |  | 1.65 (1.56-1.75) |  | 2.38 (2.04-2.78) |
|  |  |  |  |  |  |
| Abbreviations: HR: hazard rate ratio; 95% CI: 95% confidence interval.  † Adjusted for sex (binary), age (continuous), hypertension (binary), diabetes (binary), prior stroke/transient ischemic attack (binary), vascular disease (binary), and antiplatelet therapy (binary).  ║Combined endpoint of intracranial hemorrhage, gastrointestinal bleeding, extracranial or unclassified major bleeding. | | | | | |

**e-Table 6.** Hazard rate ratios of all endpoints after 1 year of follow-up (using different adjusted models), according to chronic kidney disease status (reference group: patients without chronic kidney disease).

| ENDPOINT | | Chronic kidney disease without dialysis  (CKD-no RRT) | | Chronic kidney disease with dialysis (CKD-RRT) | |
| --- | --- | --- | --- | --- | --- |
|  | |  |  |  |  |
| Ischemic stroke | |  |  |  |  |
|  | Crude HR (95% CI) |  | 1.65 (1.36-2.02) |  | 0.90 (0.45-1.81) |
|  | Adjusted HR, model 1 (age+sex only) (95% CI) |  | 1.60 (1.31-1.95) |  | 0.98 (0.49-1.97) |
|  | Adjusted HR, model 2 (hypertension) (95% CI) |  | 1.44 (1.18-1.76) |  | 0.86 (0.43-1.72) |
|  | Adjusted HR, model 3 (diabetes) (95% CI) |  | 1.43 (1.17-1.75) |  | 0.93 (0.46-1.86) |
|  | Adjusted HR, model 4 (prior stroke) (95% CI) |  | 1.37 (1.12-1.68) |  | 0.77 (0.39-1.56) |
|  | Adjusted HR, model 5 (vascular disease) (95% CI) |  | 1.56 (1.28-1.90) |  | 0.96 (0.48-1.92) |
|  | Adjusted HR, model 6 (antiplatelet therapy) (95% CI) |  | 1.61 (1.32-1.96) |  | 0.87 (0.41-1.82) |
|  | Main adjusted model (95% CI) |  | 1.24 (1.01-1.53) |  | 0.61 (0.29-1.28) |
|  |  |  |  |  |  |
| Intracranial hemorrhage | |  |  |  |  |
|  | Crude HR (95% CI) |  | 1.93 (1.00-3.71) |  | 5.71 (2.10-15.55) |
|  | Adjusted HR, model 1(age+sex only) (95% CI) |  | 1.85 (0.96-3.57) |  | 5.86 (2.14-16.01) |
|  | Adjusted HR, model 2 (hypertension) (95% CI) |  | 1.71 (0.88-3.33) |  | 5.26 (1.90-14.57) |
|  | Adjusted HR, model 3 (diabetes) (95% CI) |  | 2.06 (1.06-4.04) |  | 6.16 (2.25-16.87) |
|  | Adjusted HR, model 4 (prior stroke) (95% CI) |  | 1.72 (0.89-3.31) |  | 5.20 (1.90-14.25) |
|  | Adjusted HR, model 5 (vascular disease) (95% CI) |  | 1.92 (1.00-3.70) |  | 6.09 (2.23-16.68) |
|  | Adjusted HR, model 6 (antiplatelet therapy) (95% CI) |  | 1.88 (0.98-3.62) |  | 5.75 (2.10-15.73) |
|  | Main adjusted model (95% CI) |  | 1.86 (0.94-3.66) |  | 5.02 (1.80-14.02) |
|  |  |  |  |  |  |
| Major bleeding║ | |  |  |  |  |
|  | Crude HR (95% CI) |  | 1.53 (1.30-1.81) |  | 3.06 (2.24-4.19) |
|  | Adjusted HR, model 1 (age+sex only) (95% CI) |  | 1.48 (1.26-1.75) |  | 3.33 (2.43-4.56) |
|  | Adjusted HR, model 2 (hypertension) (95% CI) |  | 1.38 (1.17-1.64) |  | 3.05 (2.22-4.19) |
|  | Adjusted HR, model 3 (diabetes) (95% CI) |  | 1.39 (1.17-1.64) |  | 3.22 (2.35-4.41) |
|  | Adjusted HR, model 4 (prior stroke) (95% CI) |  | 1.46 (1.24-1.72) |  | 3.26 (1.24-1.72) |
|  | Adjusted HR, model 5 (vascular disease) (95% CI) |  | 1.48 (1.25-1.75) |  | 3.33 (2.43-4.56) |
|  | Adjusted HR, model 6 (antiplatelet therapy) (95% CI) |  | 1.48 (1.25-1.74) |  | 3.34 (2.44-4.57) |
|  | Main adjusted model (95% CI) |  | 1.31 (1.10-1.56) |  | 2.96 (2.16-4.07) |
|  |  |  |  |  |  |
| All-cause death | |  |  |  |  |
|  | Crude HR (95% CI) |  | 1.83 (1.71-1.96) |  | 1.59 (1.31-1.94) |
|  | Adjusted HR, model 1 (age+sex only) (95% CI) |  | 1.75 (1.63-1.88) |  | 2.44 (2.00-2.97) |
|  | Adjusted HR, model 2 (hypertension) (95% CI) |  | 1.84 (1.71-1.98) |  | 2.62 (2.15-3.19) |
|  | Adjusted HR, model 3 (diabetes) (95% CI) |  | 1.69 (1.57-1.81) |  | 2.39 (1.96-2.91) |
|  | Adjusted HR, model 4 (prior stroke) (95% CI) |  | 1.72 (1.60-1.84) |  | 2.37 (1.94-2.88) |
|  | Adjusted HR, model 5 (vascular disease) (95% CI) |  | 1.74 (1.62-1.87) |  | 2.42 (1.99-2.94) |
|  | Adjusted HR, model 6 (antiplatelet therapy) (95% CI) |  | 1.78 (1.66-1.91) |  | 2.34 (1.92-2.85) |
|  | Main adjusted model (95% CI) |  | 1.72 (1.60-1.86) |  | 2.31 (1.89-2.81) |
| Abbreviations: HR: hazard rate ratio; 95% CI: 95% confidence interval.  Model 1: Adjusted for sex (binary) and age (continuous). Model 2: Adjusted for sex (binary), age (continuous), and hypertension (binary). Model 3: Adjusted for sex (binary), age (continuous), and diabetes (binary). Model 4: Adjusted for sex (binary), age (continuous), and prior stroke (binary). Model 5: Adjusted for sex (binary), age (continuous), and vascular disease (binary). Model 6: Adjusted for sex (binary), age (continuous), and antiplatelet therapy (binary).  ║Combined endpoint of intracranial hemorrhage, gastrointestinal bleeding, extracranial or unclassified major bleeding. | | | | | |

**e-Table 7.** Hazard rate ratios of all endpoints after 5 years of follow-up (using different adjusted models), according to chronic kidney disease status (reference group: patients without chronic kidney disease).

| ENDPOINT | | Chronic kidney disease without dialysis  (CKD-no RRT) | | Chronic kidney disease with dialysis (CKD-RRT) | |
| --- | --- | --- | --- | --- | --- |
|  | |  |  |  |  |
| Ischemic stroke | |  |  |  |  |
|  | Crude HR (95% CI) |  | 1.71 (1.48-1.97) |  | 1.05 (0.65-1.69) |
|  | Adjusted HR, model 1 (age+sex only) (95% CI) |  | 1.65 (1.43-1.91) |  | 1.20 (0.74-1.93) |
|  | Adjusted HR, model 2 (hypertension) (95% CI) |  | 1.51 (1.31-1.75) |  | 1.07 (0.66-1.73) |
|  | Adjusted HR, model 3 (diabetes) (95% CI) |  | 1.47 (1.27-1.70) |  | 1.13 (0.70-1.82) |
|  | Adjusted HR, model 4 (prior stroke) (95% CI) |  | 1.49 (1.29-1.72) |  | 0.96 (0.60-1.55) |
|  | Adjusted HR, model 5 (vascular disease) (95% CI) |  | 1.62 (1.40-1.87) |  | 1.17 (0.73-1.89) |
|  | Adjusted HR, model 6 (antiplatelet therapy) (95% CI) |  | 1.66 (1.44-1.92) |  | 1.13 (0.69-1.85) |
|  | Main adjusted model (95% CI) |  | 1.31 (1.13-1.52) |  | 0.80 (0.49-1.31) |
|  |  |  |  |  |  |
| Intracranial hemorrhage | |  |  |  |  |
|  | Crude HR (95% CI) |  | 1.95 (1.24-3.05) |  | 2.85 (1.06-7.68) |
|  | Adjusted HR, model 1 (age+sex only) (95% CI) |  | 1.90 (1.21-2.97) |  | 3.08 (1.14-8.31) |
|  | Adjusted HR, model 2 (hypertension) (95% CI) |  | 1.70 (1.08-2.69) |  | 2.70 (1.00-2.69) |
|  | Adjusted HR, model 3 (diabetes) (95% CI) |  | 1.85 (1.17-2.93) |  | 3.05 (1.13-8.23) |
|  | Adjusted HR, model 4 (prior stroke) (95% CI) |  | 1.78 (1.14-2.79) |  | 2.73 (1.01-7.38) |
|  | Adjusted HR, model 5 (vascular disease) (95% CI) |  | 1.94 (1.24-3.04) |  | 3.15 (1.17-8.50) |
|  | Adjusted HR, model 6 (antiplatelet therapy) (95% CI) |  | 1.91 (1.22-2.99) |  | 3.03 (1.12-8.18) |
|  | Main adjusted model (95% CI) |  | 1.66 (1.04-2.65) |  | 2.43 (0.89-6.61) |
|  |  |  |  |  |  |
| Major bleeding║ | |  |  |  |  |
|  | Crude HR (95% CI) |  | 1.49 (1.33-1.68) |  | 2.93 (2.31-3.72) |
|  | Adjusted HR, model 1 (age+sex only) (95% CI) |  | 1.46 (1.29-1.64) |  | 3.22 (2.54-4.09) |
|  | Adjusted HR, model 2 (hypertension) (95% CI) |  | 1.38 (1.22-1.56) |  | 3.02 (2.38-3.84) |
|  | Adjusted HR, model 3 (diabetes) (95% CI) |  | 1.33 (1.18-1.51) |  | 3.09 (2.43-3.92) |
|  | Adjusted HR, model 4 (prior stroke) (95% CI) |  | 1.44 (1.28-1.63) |  | 3.17 (2.50-4.03) |
|  | Adjusted HR, model 5 (vascular disease) (95% CI) |  | 1.45 (1.29-1.64) |  | 3.22 (2.53-4.08) |
|  | Adjusted HR, model 6 (antiplatelet therapy) (95% CI) |  | 1.45 (1.29-1.64) |  | 3.23 (2.55-4.10) |
|  | Main adjusted model (95% CI) |  | 1.28 (1.13-1.45) |  | 2.91 (2.29-3.70) |
|  |  |  |  |  |  |
| All-cause death | |  |  |  |  |
|  | Crude HR (95% CI) |  | 1.76 (1.67-1.86) |  | 1.70 (1.47-1.96) |
|  | Adjusted HR, model 1 (age+sex only) (95% CI) |  | 1.72 (1.63-1.82) |  | 2.59 (2.24-2.99) |
|  | Adjusted HR, model 2 (hypertension) (95% CI) |  | 1.77 (1.68-1.87) |  | 2.69 (2.32-3.11) |
|  | Adjusted HR, model 3 (diabetes) (95% CI) |  | 1.62 (1.54-1.71) |  | 2.50 (2.17-2.89) |
|  | Adjusted HR, model 4 (prior stroke) (95% CI) |  | 1.70 (1.61-1.79) |  | 2.50 (2.17-2.89) |
|  | Adjusted HR, model 5 (vascular disease) (95% CI) |  | 1.71 (1.63-1.81) |  | 2.57 (2.22-2.97) |
|  | Adjusted HR, model 6 (antiplatelet therapy) (95% CI) |  | 1.73 (1.64-1.83) |  | 2.52 (2.18-2.91) |
|  | Main adjusted model (95% CI) |  | 1.63 (1.55-1.73) |  | 2.40 (2.07-2.77) |
| Abbreviations: HR: hazard rate ratio; 95% CI: 95% confidence interval.  Model 1: Adjusted for sex (binary) and age (continuous). Model 2: Adjusted for sex (binary), age (continuous), and hypertension (binary). Model 3: Adjusted for sex (binary), age (continuous), and diabetes (binary). Model 4: Adjusted for sex (binary), age (continuous), and prior stroke (binary). Model 5: Adjusted for sex (binary), age (continuous), and vascular disease (binary). Model 6: Adjusted for sex (binary), age (continuous), and antiplatelet therapy (binary).  ║Combined endpoint of intracranial hemorrhage, gastrointestinal bleeding, extracranial or unclassified major bleeding. | | | | | |

**Methodological details.** Analysis on a risk-scale.

In order to have the complete risk assessment of chronic kidney disease in heart failure patients, both rates and relative risks are needed. Thus, regression analysis was used to compare the 1- and 5-year relative risks of the endpoints according to the presence of chronic kidney disease. For this analysis, we used generalized linear regression alongside the pseudo-value method in order to take into account the competing risks of death (Klein J, Andersen P. Regression modeling of competing risks data based on pseudovalues of the cumulative incidence function. *Biometrics*. 2005;61:223–229; Klein J, Logan B, Harhoff M, Andersen P. Analyzing survival curves at a fixed point in time. *Stat Med*. 2007;26:4505–4519). The pseudo-value regression technique reduces to simple regression (with a log-link function) on the event status indicator at 1 and 5 year in the absence of censoring, whereas censored observations (for which the event status is not observed) are replaced with pseudo-observations based upon Aalen-Johansen cumulative incidence estimates using the jackknife method.
